# RETRACTION: Effect of Right Anterolateral Thoracotomy Versus Median Sternotomy on Postoperative Wound Tissue Repair in Patients With Congenital Heart Disease: A Meta‐Analysis

**DOI:** 10.1111/iwj.70382

**Published:** 2025-03-21

## Abstract

**RETRACTION:** HeR. , ZhangK. , ZhouC. , and PeiC. , “Effect of Right Anterolateral Thoracotomy Versus Median Sternotomy on Postoperative Wound Tissue Repair in Patients With Congenital Heart Disease: A Meta‐Analysis,” International Wound Journal 21, no. 1 (2024): e14343, 10.1111/iwj.14343.37641209 PMC10781613

The above article, published online on 28 August 2023, in Wiley Online Library (http://onlinelibrary.wiley.com/), has been retracted by agreement between the journal Editor in Chief, Professor Keith Harding; and John Wiley & Sons Ltd. Following an investigation by the publisher, all parties have concluded that this article was accepted solely on the basis of a compromised peer review process. The editors have therefore decided to retract the article. The authors did not respond to our notice regarding the retraction.

**RETRACTION:** R. He , K. Zhang , C. Zhou , and C. Pei , “Effect of Right Anterolateral Thoracotomy Versus Median Sternotomy on Postoperative Wound Tissue Repair in Patients With Congenital Heart Disease: A Meta‐Analysis,” International Wound Journal 21, no. 1 (2024): e14343, 10.1111/iwj.14343.37641209 PMC10781613

The above article, published online on 28 August 2023, in Wiley Online Library (http://onlinelibrary.wiley.com/), has been retracted by agreement between the journal Editor in Chief, Professor Keith Harding; and John Wiley & Sons Ltd. Following an investigation by the publisher, all parties have concluded that this article was accepted solely on the basis of a compromised peer review process. The editors have therefore decided to retract the article. The authors did not respond to our notice regarding the retraction.

